# The Effect of Altering Body Posture and Barbell Position on the Between-Session Reliability of Force-Time Curve Characteristics in the Isometric Mid-Thigh Pull

**DOI:** 10.3390/sports6040162

**Published:** 2018-11-30

**Authors:** Stuart N. Guppy, Claire J. Brady, Yosuke Kotani, Michael H. Stone, Nikola Medic, Guy Gregory Haff

**Affiliations:** 1 Centre for Exercise and Sports Science Research, Edith Cowan University, Joondalup 6027, Australia; y.kotani@ecu.edu.au (Y.K.); n.medic@ecu.edu.au (N.M.); g.haff@ecu.edu.au (G.G.H.); 2 Department of Physical Education & Sports Sciences, University of Limerick, Limerick V94 T9PX, Ireland; Claire.Brady@ul.ie; 3 Centre of Excellence for Sports Science and Coach Education, Department of Exercise and Sport Science, East Tennessee State University, Johnson City, TN 37614, USA; stonem@etsu.edu

**Keywords:** strength testing, maximum force, rate of force development, impulse, performance testing

## Abstract

Seventeen strength and power athletes (*n* = 11 males, 6 females; height: 177.5 ± 7.0 cm, 165.8 ± 11.4 cm; body mass: 90.0 ± 14.1 kg, 66.4 ± 13.9 kg; age: 30.6 ± 10.4 years, 30.8 ± 8.7 years), who regularly performed weightlifting movements during their resistance training programs, were recruited to examine the effect of altering body posture and barbell position on the between-session reliability of force-time characteristics generated in the isometric mid-thigh pull (IMTP). After participants were familiarised with the testing protocol, they undertook two testing sessions which were separated by seven days. In each session, the participants performed three maximal IMTP trials in each of the four testing positions examined, with the order of testing randomized. In each position, no significant differences were found between sessions for all force-time characteristics (*p* = >0.05). Peak force (PF), time-specific force (F50, F90, F150, F200, F250) and IMP time-bands (0–50, 0–90, 0–150, 0–200, 0–250 ms) were reliable across each of the four testing positions (ICC ≥ 0.7, CV ≤ 15%). Time to peak force, peak RFD, RFD time-bands (0–50, 0–90, 0–150, 0–200, 0–250 ms) and peak IMP were unreliable regardless of the testing position used (ICC = <0.7, CV = >15%). Overall, the use of body postures and barbell positions during the IMTP that do not correspond to the second pull of the clean have no adverse effect on the reliability of the force-time characteristics generated.

## 1. Introduction

Isometric tests, such as the isometric mid-thigh pull (IMTP), enable efficient assessment of skeletal muscle function in athletic populations. As isometric tests produce a force-time curve, it is possible to assess multiple physical characteristics underpinning sports performance, such as force generating capacity, rate of force development (RFD) and impulse (IMP) within a single trial [1,2]. Furthermore, when performed in conjunction with traditional dynamic measures of strength (one repetition maximum squat, deadlift and bench press) [3,4] or power (jumping and throwing movements) [5], the use of isometric tests may provide a clearer assessment of changes induced by training interventions than relying on a single dynamic measure [6]. While single-joint laboratory-based isometric tests, such as knee extension or plantar-flexion, typically display poor relationships to dynamic multi-joint movements commonly found in sport [7,8,9], multi-joint isometric tests such as the IMTP have been shown to display strong relationships to dynamic athletic performance [10,11,12] and are therefore commonly used in applied sport settings.

Originally designed, in part, as a monitoring tool for physical characteristics underpinning successful performance in weightlifting, the testing position used during the performance of the IMTP closely corresponds to the second pull position found in the clean [13,14]. Importantly, during dynamic performance of the weightlifting movements, this position results in the highest barbell velocity and force output [15,16,17]. Due to this mechanical similarity between the isometric and dynamic positions, peak force (PF) in the IMTP is highly correlated with performance in both the clean & jerk (r = 0.84) and snatch (r = 0.83), along with competition total (r = 0.84) [18]. Furthermore, PF is correlated with performance in derivatives of the weightlifting movements such as the power clean (r = 0.57–0.67) [19] and power snatch (r = 0.94–0.98) [20]. In addition to its use in weightlifting as a monitoring tool, the IMTP has been used in several other sports [21,22,23], with both force and RFD characteristics being related to performance in common sporting movements such as sprinting [10], jumping [11], throwing [20] and change of direction [10], along with being used as a fatigue monitoring tool in tennis [24]. 

Because of the increasingly common use of the IMTP as an assessment tool within comprehensive athlete monitoring programs it is important that the force-time characteristics used to assess the athletes’ skeletal muscle function are reliable and therefore provide accurate information about the effect of both competition and training upon an athlete’s physical condition [25]. Previous research by Haff et al. [26] has demonstrated that PF, time-specific force and RFD time-bands are reliable in the IMTP when using a body posture and barbell position that matches the position originally described by Haff et al. [13]. Peak RFD (pRFD), however, was unreliable unless calculated using a 20 ms sampling window [26]. Conversely, Brady et al. [27], using the same testing position as Haff et al. [26], reported that both RFD time-bands up to and including 0–150 ms and pRFD calculated in all sampling windows (2, 5, 19, 20, 30, 50 ms) are unreliable. Therefore, based on the current literature it remains unclear whether RFD time-bands are reliable methods for assessing skeletal muscle function in the IMTP.

Furthermore, as a result of the increasingly common use of the IMTP as an assessment tool, the body postures and barbell positions reported in the scientific literature have deviated substantially from the positions originally described by Haff et al. [13]. McGuigan et al. [28] reported the use of a barbell position that varied based on the athlete attaining a standardised knee angle of 130°, while Comfort et al. [29] reported the use of a barbell position that was the mid-point between the middle of the patella and the iliac crest, a position subsequently used by Wang et al. [30]. Comfort et al. [29] demonstrated that the between-session reliability of measurements of PF, maximum RFD (mRFD) using a 1.67 ms sampling window and impulse at 100 (IMP_100_), 200 (IMP_200_) and 300 (IMP_300_) ms is unaffected by changes in body posture provided the barbell position is maintained at the mid-point between the middle of the patella and the iliac crest. Subsequently however, Thomas et al. [31] reported small but significant differences in the IMP_100_, IMP_200_ and IMP_300_ generated between sessions, conflicting with the results of Comfort et al. [29]. 

While Comfort et al. [29] reported that pRFD calculated using a 1.67 ms sampling window is reliable both within- and between-session, pRFD calculated using both a 1 and 2 ms sampling window has been demonstrated as unreliable within-session by both Haff et al. [26] and Brady et al. [27]. This therefore makes comparing the between-session reliability of pRFD calculated using a 1.67 ms sampling window difficult, as there is a distinct possibility that the pRFD values calculated in the two discrete testing sessions are unreliable. Furthermore, Beckham et al. [32] reported that during trials utilising the barbell position and body posture reported by Comfort et al. [29], the subjects displayed considerable changes in body posture upon force application. It is plausible that this change in position during the test may adversely affect the reliability of the force-time characteristics produced in those testing positions, particularly time-specific force, RFD and impulse (IMP) which are sensitive to joint angle changes during trials [1]. RFD time-bands, which are potentially more reliable than pRFD measures [26,27], were not reported by Beckham et al. [32] or Comfort et al. [29] and therefore the effect of altering body posture on the reliability of these RFD variables remains unknown to date.

Therefore, the main purpose of this study was to determine the effect of altering the body posture and barbell position used during the performance of the IMTP on the between-session reliability of force-time characteristics produced during maximal trials. We hypothesised that an IMTP testing position that matched the body posture and barbell position found at the initiation of the second pull of the clean would result in reliable measures of force, RFD and impulse, while altering this position would result in unreliable measures.

## 2. Materials and Methods

### 2.1. Participants

Seventeen strength-power athletes (*n* = 11 males, 6 females; height: 177.5 ± 7.0 cm, 165.8 ± 11.4 cm; body mass: 90.0 ± 14.1 kg, 66.4 ± 13.9 kg; age: 30.6 ± 10.4 years, 30.8 ± 8.7 years) with more than 6 months of training experience in the clean (1RM: 118.5 ± 20.6 kg, 77.5 ± 10.4 kg) volunteered to undertake the experimental protocol, however one participant substantially altered their body position during all trials and therefore was excluded on the basis that data collected did not accurately reflect force-time characteristics in each of the four positions. Participants were instructed to perform no training the day prior to testing. All participants read and signed informed consent forms prior to participation in the study as required by the Edith Cowan University Human Research Ethics Committee (Project 16377).

### 2.2. Experimental Approach to the Problem

A randomised and counter-balanced testing protocol was utilised to evaluate the effects of altering barbell position and hip- and knee-angles upon the reliability of force-time characteristics produced in the IMTP. Participants undertook three testing sessions, with the first serving to familiarise them with the experimental protocol and for the collection of anthropometric data (height, body mass, right femur length), barbell height and grip width. The subsequent two testing sessions were then conducted 7 days apart, with participants performing three maximal effort IMTPs in each of the four positions presented in Table 1 during each session. 

### 2.3. Warm-Up Procedures

Prior to commencing maximal IMTP testing, participants performed a dynamic warm up that included performance of dynamic mid-thigh pulls (1 set of 3 repetitions) at 40%, 60% and 80% of their 1RM clean [29]. Once the dynamic warm-up was completed, the participants performed two submaximal IMTP’s at 50% and 75% of perceived maximal effort.

### 2.4. Isometric Mid-Thigh Pull Testing

Once the warm-up had been completed, participants were placed in the first testing position, with the order of position used randomised. Hip- and knee-angles were confirmed using hand-held goniometry. Two different barbell positions were used, with the first, termed ‘TRAD,’ corresponding to the second pull position during the clean as described by Haff et al. [26]. The second, termed ‘MT,’ corresponded to the mid-point between the iliac crest and the middle of the patella as outlined by Comfort et al. [29]. During IMTP trials using the ‘MT’ barbell position, the immovable barbell was required to cover a tape line placed on the participant’s right leg at the mid-point between the iliac crest and patella. Two different combinations of hip- and knee-angles were used for each barbell position based upon body postures reported within the literature [13,29,32]. Both an upright (TRAD 1 & MT 2) and inclined (TRAD 2 & MT 1) torso position were assessed in each barbell position. The specific combinations of hip- and knee-angles found in each of the four testing positions assessed are outlined in Table 1.

Participants were secured to the immovable barbell using weightlifting straps to prevent their hands from slipping during maximal trials [18]. All trials were performed in a custom-designed power rack (Fitness Technology, Adelaide, Australia), which enables the barbell to be positioned at any height through a combination of pins and hydraulic jacks, whilst standing on a force-plate (BP21001200, AMTI, Newton, MA, USA), sampling at 1000 Hz. Vertical ground reaction forces were collected via a BNC-2090 interface box with an analogue-to-digital card (NI-6014, National Instruments, Austin, TX, USA). Once positioned, participants performed 3 maximal IMTPs in each of the four positions. Each trial was separated by 1 min of rest and each position was separated by 2 min of rest. Prior to testing, participants were instructed to “*pull as hard and as fast as possible*” and strong verbal encouragement was provided to ensure this occurred [33]. Trials began after a countdown “3, 2, 1, Pull” with participants applying maximum effort for 5 s or until the force trace had visually declined, whichever occurred first. If there was a difference in recorded PF of greater than 250 N between trials [34] or a countermovement was visually obvious during real-time observation of the stable force trace established immediately prior to trial initiation, that trial was excluded and an additional trial was performed [23,35].

### 2.5. Isometric Force-Time Curve Analysis

All collected force-time curves were analysed using custom LabVIEW software (Version 14.0, National Instruments). The onset of force application for each trial was determined visually, with this method chosen over automated methods as it has been performed previously in the literature relating to the IMTP [32] and is suggested as the gold standard method for force onset detection in isometric trials [36,37]. After analysis, the average value of each force-time characteristic generated across the three trials was calculated for each position in a custom Excel spreadsheet (Microsoft, Redmond, WA, USA). The maximum force generated during each IMTP trial was reported as the PF. Additionally, force at 50, 90, 150, 200 and 250 milliseconds (ms) from the initiation of the pull was calculated for each trial. pRFD using a 20 ms sampling window, RFD time-bands (0–50 ms, 0–90 ms, 0–150 ms, 0–250 ms), peak IMP (pIMP) and IMP time-bands (0–50 ms, 0–90 ms, 0–150 ms, 0–200 ms, 0–250 ms) were also calculated utilising the methods outlined by Haff et al. [26] and Enoka [38] respectively. Body weight was included in the calculation of all force-time variables, allowing comparison to force-time characteristics previously presented within the literature [18].

### 2.6. Statistical Analysis

Paired comparisons (*p* < 0.05) were performed in conjunction with a Holm’s Sequential Bonferroni correction for controlling Type I error [39] to determine if significant differences existed between force-time variables produced during each testing session. Reliability of each force-time variable was assessed by determining the intraclass correlation coefficient (ICC), coefficient of variation (CV) and 95% confidence interval (CI) of log-transformed data in an Excel spreadsheet [40]. Reliability was deemed acceptable at an ICC ≥ 0.7 and a CV ≤ 15% [41]. Both typical error (TE) [40] and smallest worthwhile change (SWC) were calculated for all reliable force-time characteristics in each of the four testing positions. The SWC was determined by multiplying the between-subject SD by 0.2 (SWC_0.2_) [42], which is a small effect, or 0.5 (SWC_0.5_) [43], which is a moderate effect.

## 3. Results

**Figure 1:**
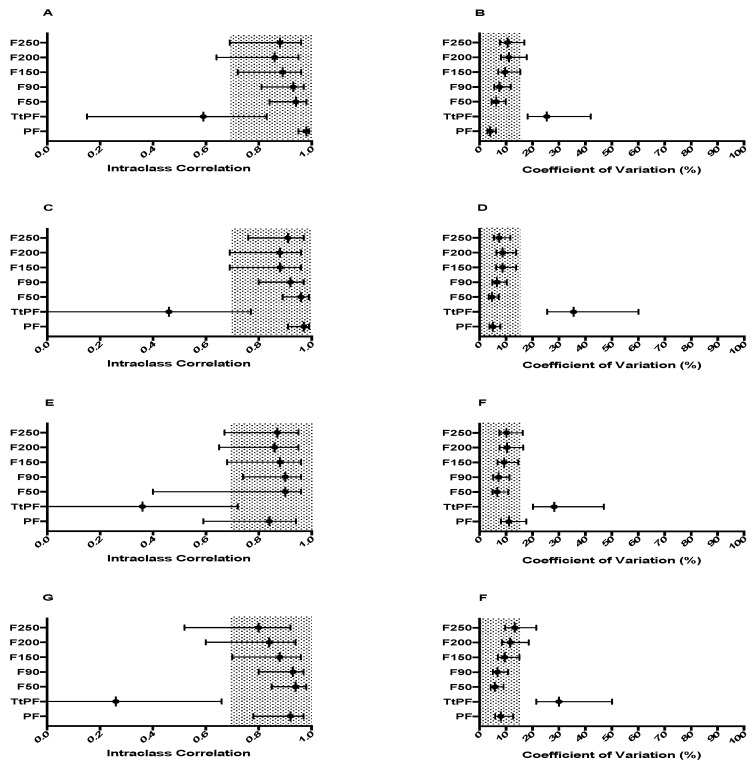
Between-session reliability statistics for force characteristics for each of the four testing positions. Grey shaded areas represented acceptable reliability (ICC ≥ 0.7, CV ≤ 15%), error bars indicate 95% confidence limits. (**A**) ICC force characteristics in TRAD 1, (**B**) CV (%), (**C**) ICC force characteristics in TRAD 2, (**D**) CV (%), (**E**) ICC force characteristics in MT 1, (**F**) CV (%), (**G**) ICC force characteristics in MT 2, (**H**) CV (%). PF, peak force; TtPF, time to peak force; F50, force at 50 ms; F90, force at 90 ms; F150, force at 150 ms; F200, force at 200 ms; F250, force at 250 ms.

**Figure 2:**
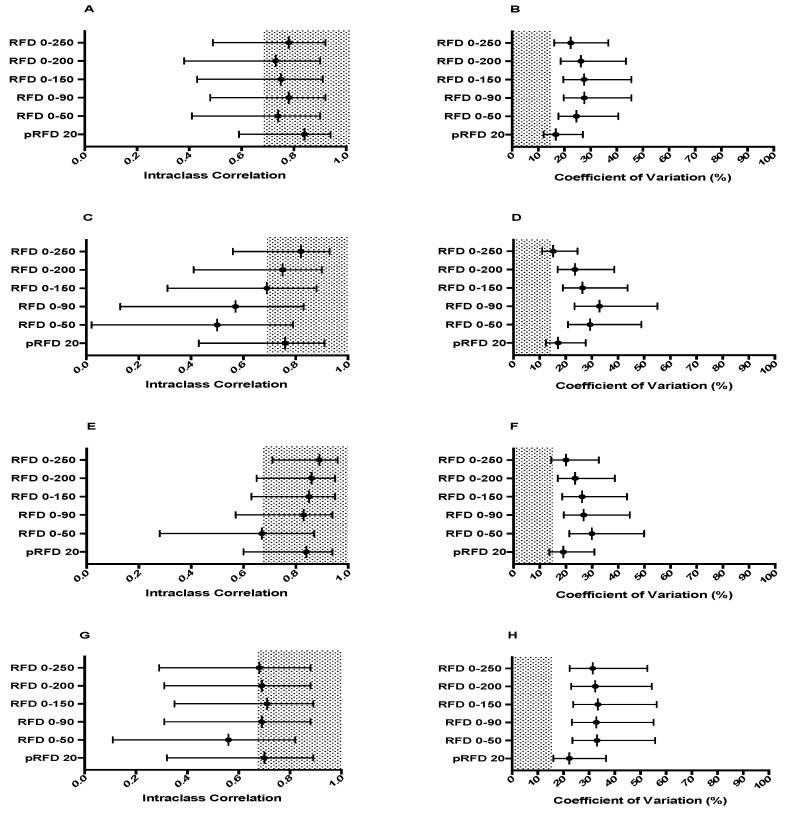
Reliability measure of the ICCs and CVs of the RFD characteristics generated in each of the four testing positions. Grey shaded areas represented acceptable reliability (ICC ≥ 0.7, CV ≤ 15%), error bars indicate 95% confidence limits. (**A**) ICC RFD characteristics in TRAD 1, (**B**) CV (%), (**C**) ICC RFD characteristics in TRAD 2, (**D**) CV (%), (**E**) ICC RFD characteristics in MT 1, (**F**) CV (%), (**G**) ICC RFD characteristics in MT 2, (**H**) CV (%). pRFD indicates peak RFD; pRFD 20, pRFD 20 ms sampling window; RFD 0–50, RFD 0–50 ms sampling window; RFD 0–90, RFD 0–90 ms sampling window; RFD 0–150, RFD 0–150 ms sampling window; RFD 0–200, RFD 0–200 ms sampling window; RFD 0–250, RFD 0–250 ms sampling window.

**Figure 3:**
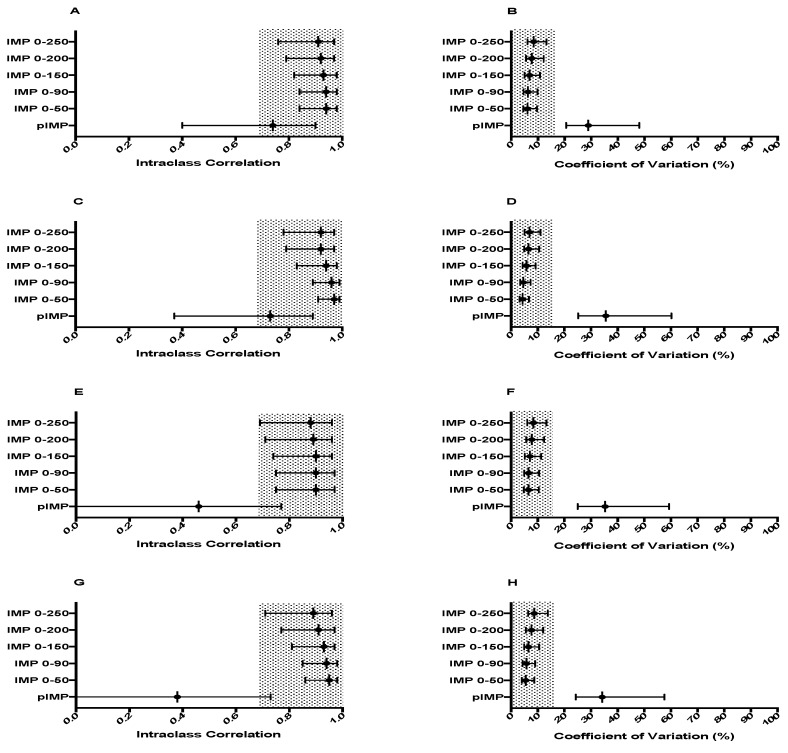
Between-session reliability measure of the ICCs and CVs of the IMP characteristics generated in each of the four testing positions. Grey shaded areas represented acceptable reliability (ICC ≥ 0.7, CV ≤ 15%), error bars indicate 95% confidence limits. (**A**) ICC IMP characteristics in TRAD 1, (**B**) CV (%), (**C**) ICC IMP characteristics in TRAD 2, (**D**) CV (%), (**E**) ICC IMP characteristics in MT 1, (**F**) CV (%), (**G**) ICC IMP characteristics in MT 2, (**H**) CV (%). pIMP, peak impulse; IMP 0–50, IMP 0–50 ms sampling window; IMP 0–90, IMP 0–90 ms sampling window; IMP 0–150, IMP 0–150 ms sampling window; IMP 0–200, IMP 0–200 ms sampling window; IMP 0–250, IMP 0–250 ms sampling window.

No significant differences were found between sessions for any force-time variable produced in each of the four positions. Figure 1 shows the reliability of all force measures. PF and all time-specific force characteristics were deemed reliable in each position. Time to peak force (TtPF) did not meet either criteria in any position. The reliability statistics for both RFD and IMP variables are shown in Figure 2 and Figure 3 respectively. pRFD and all RFD time-bands were unreliable in each of the four conditions. Similarly, peak impulse (pIMP) was unreliable across all four conditions. All IMP time-bands however, were reliable regardless of testing position used. Descriptive statistics for force-time characteristics that met the required reliability criteria are shown in Table 2 for TRAD 1, Table 3 for TRAD 2, Table 4 for MT 1 and Table 5 for MT 2.

## 4. Discussion

The primary finding of this study was that, regardless of the body and barbell position utilised during performance of the IMTP, RFD, both peak and time-specific, are not reliable measures of skeletal muscle function. Furthermore, the data suggested that the use of specific time-bands for the determination of IMP was more reliable than utilising a peak value, while force output was entirely unaffected by alterations in barbell position and body posture. The results in this study provide further insight into the correct methodology of assessment for skeletal muscle function using the IMTP.

Previously, PF measured in the IMTP has been shown to be a highly reliable measure, with ICC and CV values of 0.96–0.99 and 1.7–4.3% respectively [10,11,26,27,32]. This study reports similar ICC and CV values regardless of testing position used (See Table 2, Table 3, Table 4 and Table 5). Haff et al. [26] reported that force at 30, 50, 90, 100, 150, 200 and 250 ms demonstrated very high ICC (0.99) and very low CV values (2.3–2.7%) when utilising a barbell and body position matching the second pull of the clean, while Beckham et al. [32] similarly reported that force at 50, 90, 200 and 250 ms are reliable in the same position. This study reports similar reliability results for time-specific force to both Haff et al. [26] and Beckham et al. [32], with time-specific force at all recorded epochs reliable in each testing position. Furthermore, TtPF demonstrated poor reliability across all testing positions examined in this study. 

While the force outputs generated in this study are equally reliable in all testing positions, the current literature suggests that the magnitude of the force output generated is substantially affected by changes in position [32,44,45]. Although Comfort et al. [29] reported no significant differences between the force-time characteristics generated in nine differing combinations of hip- and knee-angles or a self-selected body posture, provided the barbell was maintained at the mid-point between the iliac crest and patella, both Beckham et al. [44] and Beckham et al. [32] have reported that a position that matches the second pull of the clean produces significantly greater force outcomes than a lower barbell position with a concurrently inclined body posture. Similarly, Dos’Santos et al. [45] reported that utilising an upright torso angle of 145° results in significantly greater force and RFD variables than a reclined torso angle of 175°, while using a barbell position that matches the second pull of the clean. Therefore, based upon the results of this study and the available literature, altering the testing position used for the IMTP does not affect the reliability of peak and time-specific force variables generated, though altering body posture away from the posture found during the second pull of the clean and adopting a lower barbell position may reduce the magnitude of those force variables generated and therefore present a less accurate assessment of the athlete’s true physical capabilities. The consistent preferential use of a position that matches the second pull of the clean will aid in the improvement of the reliability of force-time characteristics generated and provide the ability for strength and conditioning professionals to accurately compare their athletes to other populations if they wish [35].

Similar to time-specific force, previous research has suggested that RFD in specific time-bands are reliable within-session in the IMTP, with Haff et al. [26] reporting within-session ICC and CV values of >0.7 and <15% for time-bands of 0–30 ms, 0–50 ms, 0–90 ms, 0–150 ms, 0–200 ms and 0–250 ms. Furthermore, Comfort et al. [29] reported that pRFD was reliable between sessions, regardless of the body posture adopted during trials. This study however found that regardless of the position used during performance of the IMTP, CV values for all RFD time-bands were above both the 10% limit of Brady et al. [27] and the 15% limit used by both this study and Haff et al. [26]. Furthermore, pRFD calculated using a 20 ms sampling window was also deemed unreliable as while the ICC meet the required limit in all positions, the CV was >15% regardless of position. This conflicts with the results of Comfort et al. [29], who reported that pRFD calculated within a 1.67 ms sampling window was reliable between testing sessions regardless of the body posture used. Both Haff et al. [26] and Brady et al. [27] however have reported that both a 1 and 2 ms sampling window are unreliable within-session. As such, when the within-session reliability results of Haff et al. [26] and Brady et al. [27] are taken in conjunction with the between-session reliability results of the current study it does appear that pRFD, regardless of sampling window, body posture and barbell position used, should be used with caution when assessing skeletal muscle function. Strength and conditioning professionals should assess the reliability of force-time characteristics generated by their specific population to determine their suitability for use in assessing skeletal muscle function.

It is unclear why differences in the reliability of RFD characteristics have been found between the subject groups of Brady et al. [27], Comfort et al. [29] and Haff et al. [26] and this study. One potential explanation may be the differing levels of familiarisation present in the three studies [1]. Beckham et al. [46] reported that a single familiarisation session of four submaximal IMTP efforts was adequate to optimise force production, however as RFD was not examined it is unclear whether the same amount of familiarisation is sufficient to optimise RFD characteristics. While the IMTP appears to require less familiarisation to optimise force characteristics than other comparable isometric multi-joint tests like the isometric squat [46,47], it is still unclear within the current body of scientific literature the volume of pre-testing familiarisation required to optimise the reliability of RFD characteristics. As such, further research in this area is required. Furthermore, in contrast to the participants in this study, the participants in Haff et al. [26] (collegiate volleyball players) regularly performed the IMTP as part of their athlete monitoring program. As a result, those participants may possess a considerably greater ability to rapidly produce force isometrically due to the learning effect [1] than the participants examined by Brady et al. [27] and in this study, who were generally performing the test for the first time during the familiarisation session. It is therefore a plausible suggestion that RFD characteristics generated in the IMTP require substantially greater amounts of familiarisation when compared to force characteristics to produce reliable results [1] and may also explain the disparity in the reliability reported between studies. 

Unlike RFD time-bands, this study found that IMP time-bands are highly reliable between-sessions regardless of position (ICC = 0.92–0.97, CV = 4–8.7%), which aligns with the results of Comfort et al. [29] who reported that IMP_100_, IMP_200_ and IMP_300_ are reliable both within and between testing sessions. Brady et al. [27] however reported that IMP_100_, IMP_200_ and IMP_250_ was unreliable within-session when using an IMTP testing position that matched the second pull of the clean, though IMP_300_ was deemed reliable. Furthermore, unlike the current study which found no significant differences between the IMP characteristics generated in each session, Thomas et al. [31] reported small significant differences between-sessions in the IMP generated at 100, 200 and 250 ms, therefore rendering these IMP characteristics unreliable. Similar to the unknown cause for the differences in the reliability of RFD characteristics reported by different subject populations despite the use of the same IMTP protocol, it is unclear why studies have reported different reliability statistics for time-specific IMP characteristics. A potential reason is, that like both force and RFD characteristics, it is possible the level of familiarisation with the IMTP possessed by the participants may affect the reliability of IMP characteristics. It is plausible that participants with greater experience performing the IMTP and/or dynamic weightlifting movements initiated from the second pull position generate more reliable force-time characteristics overall when compared to participants with a lesser degree of experience in both the IMTP and dynamic weightlifting movements initiated from the second pull position. 

Of particular note in this study was the exclusion of one participant due to excessive changes in body posture upon the initiation of trials. While Beckham et al. [32] demonstrated that a small amount of hip and knee extension is unavoidable during IMTP trials regardless of the use of pre-tension to reduce slack in the “system,” the participant in this study shifted from an inclined torso position (125°) to a near vertical upright torso position upon trial initiation, in a manner similar to the re-positioning that occurs during the transition from the first to second pull in the weightlifting movements. Beckham et al. [32] reported a similar occurrence, with one participant changing position in a similar fashion to the participant in this study. Furthermore, Beckham et al. [32] reported that a further two participants were unable to attain the required position as described by Comfort et al. [29]. These changes in torso angle and the inability of some participants to attain the correct body posture, although rare, suggest that the initial starting position of the IMTP should be solely determined by the anthropometrics of the individual athlete, not a single standardized set of hip and knee angles that each individual in the testing cohort are forced to adopt.

There are some limitations that should be taken into consideration when examining the practicality of the results of this study. First, the participant population is somewhat homogenous in that they were all strength-power athletes with experience in the weightlifting movements. Athletes who are less experienced in the weightlifting movements, who compete in other sporting disciplines such as track and field, or compete in field-based sports (i.e., rugby, soccer and hockey) may display differing abilities in producing reliable force-time characteristics. Second, while the participants were visually monitored for changes in body posture upon force application, no direct video analysis of movement changes were monitored. It is therefore unknown the degree to which the participants who took part in this study altered their body position, which may adversely affect the reliability of force-time characteristics in isometric tests [1]. Finally, while the visual identification of force-onset has been suggested by some as the optimal method in isometric trials [36,37], recent research has suggested that the use of algorithm- or threshold-based automatic detection methods may be superior [48,49]. However, as yet, only limited research has examined this topic and further research should be performed to assess the effect of automated force-onset detection methods on the reliability of force-time characteristics.

## 5. Conclusions

The results of this study suggest that regardless of testing position used during the IMTP, both PF and time-specific force are reliable tools for the assessment of athlete’s skeletal muscle function. Conversely, the use of pRFD and RFD time-bands as monitoring or diagnostic tools should be done with caution as unreliable results may occur if the participants are not highly experienced with the testing protocol and dynamic weightlifting movements initiated from the mid-thigh position. Strength and conditioning professionals should therefore consider the preferential use of IMP time-bands in place of RFD characteristics, as IMP time-bands demonstrate a high degree of between-session reliability that is unaffected by deviations in testing position. Considering that IMP is strongly related to sprinting, jumping and change of direction ability, the use of IMP time-bands may provide superior diagnostic information to practitioners when compared to RFD variables. Further research examining these relationships across a wide range of sports should be undertaken however, as the evidence presently available within the literature specifically examining the relationships between IMP in the IMTP and markers of athletic performance is limited. The use of pIMP as an assessment and/or diagnostic tool however should be avoided as the results of this study suggested it is highly unreliable, regardless of the testing position used.

## Figures and Tables

**Table 1:** Barbell position and hip- and knee-angles for each of the four experimental positions.

Condition	Barbell Position	Knee Angle	Hip Angle
TRAD 1	Traditional	~145°	~145°
TRAD 2	Traditional	~145°	120°
MT 1	Mid-Thigh	120°	125°
MT 2	Mid-Thigh	120°	145°

**Table 2:** Descriptive and reliability statistics for force-time characteristics generated in TRAD 1 that demonstrate acceptable reliability.

Variable	Mean ± SD T1	Mean ± SD T2	ICC	95% CI		CV (%)	95% CI		TE	SWC_0.2_	SWC_0.5_
				Lower	Upper		Lower	Upper			
PF (N)	2748.38 ± 730.00	2728.23 ± 643.83	0.98	0.95	0.99	4.00	2.90	6.2	98.04	247.77	686.92
F_50_ (N)	1010.56 ± 255.70	965.59 ± 204.45	0.94	0.84	0.98	6.30	4.60	9.90	76.31	92.03	230.07
F_90_ (N)	1173.34 ± 313.03	1094.63 ± 246.31	0.93	0.81	0.97	7.50	5.50	11.80	100.02	111.87	279.67
F_150_ (N)	1456.34 ± 403.36	1348.40 ± 318.06	0.89	0.72	0.96	9.70	7.10	15.40	140.39	144.28	360.71
F_200_ (N)	1670.12 ± 454.14	1537.89 ± 360.31	0.86	0.64	0.95	11.10	8.10	17.80	179.20	162.89	407.23
F_250_ (N)	1877.53 ± 510.16	1740.23 ± 422.69	0.88	0.69	0.96	10.60	7.70	16.90	195.65	186.57	466.42
IMP_0–50_ (Ns)	47.85 ± 11.94	46.36 ± 9.64	0.94	0.84	0.98	6.10	4.50	9.70	3.54	4.32	10.79
IMP_0–90_ (Ns)	91.40 ± 23.12	87.39 ± 18.32	0.94	0.84	0.98	6.30	4.60	9.90	6.90	8.33	20.82
IMP_0–150_ (Ns)	170.58 ± 44.29	160.83 ± 35.20	0.93	0.82	0.98	6.90	5.00	10.80	13.43	15.90	39.75
IMP_0–200_ (Ns)	248.88 ± 64.96	233.15 ± 51.60	0.92	0.79	0.97	7.70	5.60	12.20	20.65	23.31	58.28
IMP_0–250_ (Ns)	337.71 ± 87.96	315.10 ± 70.18	0.91	0.76	0.97	8.40	6.10	13.20	29.27	31.63	79.07

**Table 3:** Descriptive and reliability statistics for force-time characteristics generated in TRAD 2 that demonstrate acceptable reliability.

Variable	Mean ± SD T1	Mean ± SD T2	ICC	95% CI		CV (%)	95% CI		TE	SWC_0.2_	SWC_0.5_
				Lower	Upper		Lower	Upper			
PF (N)	2117.79 ± 522.43	2130.91 ± 559.44	0.97	0.91	0.99	5.00	3.70	7.80	102.17	216.37	540.93
F_50_ (N)	851.52 ± 176.54	872.76 ± 182.69	0.96	0.89	0.99	4.70	3.40	7.30	36.95	71.85	179.61
F_90_ (N)	938.27 ± 203.84	956.65 ± 192.42	0.92	0.80	0.97	6.50	4.80	10.30	58.83	79.25	198.13
F_150_ (N)	1139.05 ± 274.06	1138.90 ± 228.50	0.88	0.69	0.96	8.70	6.30	13.80	98.52	100.51	251.28
F_200_ (N)	1297.21 ± 319.28	1283.66 ± 257.64	0.88	0.69	0.96	8.70	6.40	13.80	113.06	115.39	288.46
F_250_ (N)	1429.40 ± 338.54	1415.68 ± 284.45	0.91	0.76	0.97	7.40	5.40	11.60	103.78	124.60	311.50
IMP_0–50_ (Ns)	41.33 ± 8.58	42.33 ± 9.05	0.97	0.91	0.99	4.30	3.20	6.70	1.61	3.53	8.82
IMP_0–90_ (Ns)	77.01 ± 16.25	78.80 ± 16.44	0.96	0.89	0.99	4.60	3.40	7.30	3.30	6.50	16.25
IMP_0–150_ (Ns)	139.27 ± 30.00	141.60 ± 28.64	0.94	0.83	0.98	5.80	4.30	9.20	7.72	11.73	29.32
IMP_0–200_ (Ns)	200.37 ± 44.61	202.31 ± 40.47	0.92	0.79	0.97	6.60	4.90	10.50	12.93	17.02	42.54
IMP_0–250_ (Ns)	268.68 ± 60.85	269.86 ± 53.65	0.92	0.78	0.97	6.90	5.10	11.00	18.24	22.90	63.73

**Table 4:** Descriptive and reliability statistics for force-time characteristics generated in MT 1 that demonstrate acceptable reliability.

Variable	Mean ± SD T1	Mean ± SD T2	ICC	95% CI		CV (%)	95% CI		TE	SWC_0.2_	SWC_0.5_
				Lower	Upper		Lower	Upper			
PF (N)	2425.37 ± 689.85	2435.65 ± 498.13	0.84	0.59	0.94	11.10	8.10	17.70	268.01	237.59	593.99
F_50_ (N)	921.71 ± 187.25	951.68 ± 164.31	0.90	0.40	0.96	6.70	4.90	10.50	58.29	70.31	175.78
F_90_ (N)	1011.21 ± 229.44	1043.72 ± 187.89	0.90	0.74	0.96	7.20	5.20	11.30	69.33	83.47	208.67
F_150_ (N)	1208.21 ± 336.67	1242.34 ± 249.78	0.88	0.68	0.96	9.30	6.80	14.70	115.04	117.29	293.23
F_200_ (N)	1372.02 ± 409.76	1405.81 ± 286.19	0.86	0.65	0.95	10.40	7.60	16.50	151.13	139.19	347.98
F_250_ (N)	1521.98 ± 452.39	1559.56 ± 316.65	0.87	0.67	0.95	10.20	7.50	16.30	162.18	153.81	384.52
IMP_0–50_ (Ns)	44.76 ± 8.87	45.97 ± 7.96	0.90	0.75	0.97	6.60	4.80	10.40	2.81	3.37	8.42
IMP_0–90_ (Ns)	83.29 ± 17.05	85.74 ± 14.81	0.90	0.75	0.97	6.60	4.90	10.50	5.25	6.37	15.93
IMP_0–150_ (Ns)	149.81 ± 33.64	154.26 ± 27.30	0.90	0.74	0.94	7.10	5.20	11.30	10.30	12.19	30.47
IMP_0–200_ (Ns)	214.50 ± 52.10	220.57 ± 39.99	0.89	0.71	0.96	7.80	5.70	12.40	16.65	18.42	46.05

**Table 5:** Descriptive and reliability statistics for force-time characteristics in MT 2 that demonstrate acceptable reliability.

Variable	Mean ± SD T1	Mean ± SD T2	ICC	95% CI		CV (%)	95% CI		TE	SWC_0.2_	SWC_0.5_
				Lower	Upper		Lower	Upper			
PF (N)	2746.23 ± 717.06	2810.19 ± 672.41	0.92	0.78	0.97	8.00	5.90	12.70	217.16	277.89	694.73
F_50_ (N)	1017.54 ± 230.57	1038.56 ± 218.08	0.94	0.85	0.98	5.80	4.20	9.10	58.43	89.73	224.33
F_90_ (N)	1102.11 ± 266.12	1118.30 ± 236.44	0.93	0.80	0.97	6.80	5.00	10.80	74.53	100.51	251.28
F_150_ (N)	1294.31 ± 352.05	1311.89 ± 286.61	0.88	0.70	0.96	9.50	6.90	15.00	119.24	127.73	319.33
F_200_ (N)	1470.60 ± 426.38	1495.76 ± 327.08	0.84	0.60	0.94	11.60	8.50	18.60	167.03	150.69	376.73
F_250_ (N)	1658.70 ± 491.04	1683.30 ± 361.61	0.80	0.52	0.92	13.30	9.70	21.40	213.20	170.53	426.32
IMP_0–50_ (Ns)	49.66 ± 11.07	50.63 ± 10.66	0.95	0.86	0.98	5.50	4.10	8.70	2.79	4.35	10.87
IMP_0–90_ (Ns)	91.94 ± 20.88	93.65 ± 19.68	0.94	0.85	0.98	5.80	4.30	9.10	5.36	8.11	20.28
IMP_0–150_ (Ns)	163.72 ± 39.14	166.30 ± 35.06	0.93	0.81	0.97	6.60	4.90	10.50	7.99	14.84	37.10
IMP_0–200_ (Ns)	232.89 ± 58.25	236.52 ± 50.05	0.91	0.77	0.97	7.70	5.60	12.10	17.69	21.66	54.15
IMP_0–250_ (Ns)	311.13 ± 80.70	316.09 ± 66.65	0.89	0.71	0.96	8.70	6.40	13.80	26.92	29.47	73.68
